# Impact of *Toxoplasma gondii* Infection on Host Non-coding RNA Responses

**DOI:** 10.3389/fcimb.2019.00132

**Published:** 2019-05-14

**Authors:** Kayla L. Menard, Breanne E. Haskins, Eric Y. Denkers

**Affiliations:** Department of Biology, Center for Evolutionary and Theoretical Immunology, University of New Mexico, Albuquerque, NM, United States

**Keywords:** non-coding RNA, microRNA, miRNA, long non-coding RNA, lncRNA, *Toxoplasma gondii*, parasite

## Abstract

As an intracellular microbe, *Toxoplasma gondii* must establish a highly intimate relationship with its host to ensure success as a parasite. Many studies over the last decade-and-a-half have highlighted how the host reshapes its immunoproteome to survive infection, and conversely how the parasite regulates host responses to ensure persistence. The role of host non-protein-coding RNA during infection is a vast and largely unexplored area of emerging interest. The potential importance of this facet of the host-parasite interaction is underscored by current estimates that as much as 80% of the host genome is transcribed into non-translated RNA. Here, we review the current state of knowledge with respect to two major classes of non-coding RNA, microRNA (miRNA) and long non-coding RNA (lncRNA), in the host response to *T. gondii* infection. These two classes of regulatory RNA are known to have profound and widespread effects on cell function. However, their impact on infection and immunity is not well-understood, particularly for the response to *T. gondii*. Nevertheless, numerous miRNAs have been identified that are upregulated by *Toxoplasma*, and emerging evidence suggests a functional role during infection. While the field of lncRNA is in its infancy, it is already clear that *Toxoplasma* is also a strong trigger for this class of regulatory RNA. Non-coding RNA responses induced by *T. gondii* are likely to be major determinants of the host's ability to resist infection and the parasite's ability to establish long-term latency.

## Introduction

### *Toxoplasma gondii* and the Immune Response

*Toxoplasma gondii* is one of the most prevalent human parasites in the world. It infects a wide range of species and establishes latent infection in brain and muscle tissue. In immune compromised individuals, as well as in the developing fetus, infection can result in severe disease (McLeod et al., 2013). *Toxoplasma* initiates strong protective Th1 immunity through induction of dendritic cell IL-12, while also inducing the activity of counter-regulatory cytokines such as IL-10 (Dupont et al., 2012; Sasai et al., 2018). In mouse models, parasite profilin functions as a pathogen-associated molecular pattern molecule triggering IL-12 through host Toll-like receptors 11 and 12 (Andrade et al., 2013; Raetz et al., 2013; Gazzinelli et al., 2014; Yarovinsky, 2014). From within the cell, *Toxoplasma* directly injects host-directed effector proteins such as ROP16, TgIST, GRA18, and GRA24 (Olias et al., 2016; Hakimi et al., 2017; He et al., 2018). These proteins seize control of host signaling responses through respective activation of STAT3/6, NFκB, and p38 MAPK molecules (Ong et al., 2010; Butcher et al., 2011; Rosowski et al., 2011; Braun et al., 2013). It is likely that a major factor in the success of *Toxoplasma* lies in its ability to produce host-directed effectors that act to ensure a balance between pro-inflammatory and anti-inflammatory responses. Host non-coding RNA responses are now emerging as important regulators of cell function. Regarding the host response to *Toxoplasma*, several microRNAs and a growing list of long non-coding RNAs are known to be triggered by infection. Here, we survey the current state of our knowledge of this important but still little understood class of host responses during infection with *T. gondii*.

### Non-coding RNA

Only a small percentage (<3%) of the genome codes for proteins, yet the majority (~80%) is actively transcribed (Spurlock et al., 2016; Atianand et al., 2017). The transcripts that do not code for proteins are collectively referred to as non-coding RNAs (ncRNAs) and they are separated into two general categories: shorter non-coding RNAs and longer non-coding RNAs (Xue et al., 2017; Zhang et al., 2017). Shorter non-coding RNAs are defined as being <200 nucleotides in length and include microRNAs (miRNAs), short interfering RNAs (siRNAs), transfer RNAs (tRNAs), piwi-interacting RNAs (piwiRNA), and small nucleolar RNAs (snoRNAs). Non-coding RNAs <200 nucleotides include ribosomal RNAs (rRNAs) and long non-coding RNAs (lncRNAs). Here, we focus on two types of non-coding RNAs: microRNAs and long non-coding RNAs. While several other classes of RNAs exist, miRNAs and lncRNAs are the two major classes of host ncRNA that have been examined during *Toxoplasma* infection.

### microRNA

**Figure 1:**
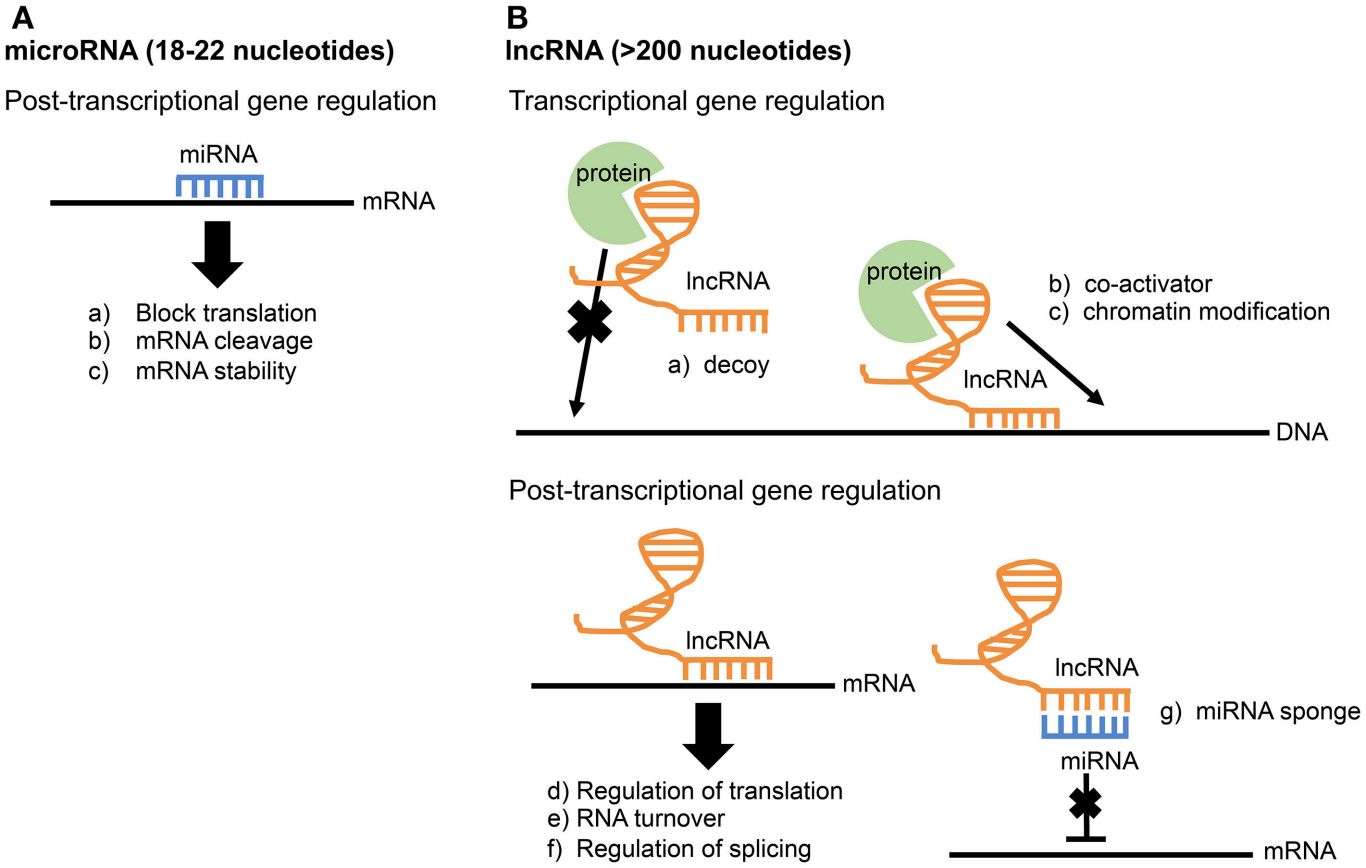
Comparison of miRNA and lncRNA function. **(A)** microRNAs function post-transcriptionally through direct base-pair interactions with mRNA. **(B)** Due to their larger size, lncRNAs have greater functional diversity and can interact with RNA, DNA, and protein. lncRNAs are known to influence gene expression at both the transcriptional and post-transcriptional level.

MicroRNAs are ~18 to 22 nucleotides in length. While examples of miRNAs acting transcriptionally exist, they primarily function post-transcriptionally by directly binding to mRNAs through direct base pair interactions (Xue et al., 2017). This interaction leads to mRNA cleavage, mRNA degradation, or blocking of translation (Figure 1A). MicroRNAs play important roles in regulating both innate and adaptive immunity. For example, the miR-17-92 cluster regulates B-cell, T-cell, and monocyte development through downregulation of the proapoptotic protein Bim (Xiao et al., 2008). The miR-146 family is a negative regulator of the innate immune response and may target TRAF6 and IRAK1 (Taganov et al., 2006; Lindsay, 2008; Cannella et al., 2014). miR-155 is a regulator of T-cell and B-cell maturation, as well as the innate immune response (Lindsay, 2008; Cannella et al., 2014).

### lncRNA

The largest group of RNA produced is long non-coding RNAs (lncRNAs), and it accounts for up to 68% of the transcriptome, not including ribosomal RNAs (Iyer et al., 2015; Chen et al., 2017). Compared to microRNAs, lncRNAs are much longer and more complex in structure and function. Thus, lncRNAs have multiple operational units and extensive functional diversity through their ability to interact with RNA, DNA, and protein (Guttman and Rinn, 2012; Fitzgerald and Caffrey, 2014; Chen et al., 2017). lncRNAs are widely involved in gene regulation at both transcriptional and post-transcriptional levels. Known functions of lncRNAs include transcriptional co-activation, recruitment of chromatin modifiers, miRNA sponges, regulation of splicing, and mRNA stabilization (Figure 1B; Fitzgerald and Caffrey, 2014; Szcześniak and Makałowska, 2016). The study of lncRNAs in the immune system is a relatively new field. In fact, the first study identifying a function for a particular lncRNA involved in the innate immune response was published as recently as 2013 (Carpenter et al., 2013). This lncRNA, lncRNA-Cox2, is broadly involved in both activation and repression of immune genes by performing functions both in *cis* at nearby genes and *trans* at genes on different chromosomes (Elling et al., 2018). In addition to lncRNA-Cox2, several other lncRNAs are now known to play a role in the immune response (Spurlock et al., 2016; Atianand et al., 2017; Chen et al., 2017). Recently, lncRNA responses were examined in epithelial cells infected with the intestinal pathogen *Cryptosporidium parvum*, an apicomplexan related to *Toxoplasma*. One lncRNA in particular, NR_045064, was found to regulate selected host defense genes through modifications in chromatin structure (Li et al., 2018).

### Host microRNA in the Response to *T. gondii*

Numerous studies have surveyed global host miRNA responses during *T. gondii* infection in different anatomical regions (liver, spleen, brain, plasma), cell types (primary human foreskin fibroblasts, neuroepitheliomal cells, peripheral blood mononuclear cell-derived macrophages), and mammalian species (mouse, human, cat, pig). Several different parasite strains and infectious stages of *T. gondii* have also been employed. These studies are summarized in Table 1. Here, we highlight some key findings as well as underscore some of the commonalities between the studies.

**Table 1:** Studies surveying host microRNAs and lncRNAs differentially expressed during *T. gondii* infection.

**Study title**	**Type**	**Host**	**Cell/tissue**	**Strain^**a**^**	**Method**	**FD^**b**^**	**References**
Plasma microRNAs are promising novel biomarkers for the early detection of *Toxoplasma gondii* infection	miRNA	Mouse	Plasma from infected mice	RH, ME49	Real-time PCR arrays	No	Jia et al., 2014
Analysis of miRNA expression profiling in mouse spleen affected by acute *Toxoplasma gondii* infection.	miRNA	Mouse	Spleen from infected mice	RH	High-throughput sequencing	No	He et al., 2016
Characterization of mouse brain microRNAs after infection with cyst-forming *Toxoplasma gondii*	miRNA	Mouse	Brain from infected mice	PRU cysts	High-throughput sequencing	No	Xu et al., 2013
*Toxoplasma gondii* infection of fibroblasts causes the production of exosome-like vesicles containing a unique array of mRNA and miRNA transcripts compared to serum starvation	miRNA	Human	Exosome-like vesicles isolated from HFF^c^	PRU	Microarray	No	Pope and Lässer, 2013
Exosomes secreted by *Toxoplasma gondii*-infected L6 cells: their effects on host cell proliferation and cell cycle changes	miRNA	Rat	Exosomes isolated from L6 myoblast cell line	RH	Microarray	No	Kim et al., 2016
Differential brain microRNA expression profiles after acute and chronic infection of mice with *Toxoplasma gondii* oocysts	miRNA	Mouse	Brain from infected mice	PRU oocysts	High-throughput sequencing	No	Hu et al., 2018
STAT3-dependent transactivation of miRNA genes following *Toxoplasma gondii* infection in macrophage	miRNA	Human	Macrophages derived from PBMCs^d^	TgCtwh3	Microarray	Yes	Cai et al., 2013
*Toxoplasma gondii* infection specifically increases the levels of key host microRNAs	miRNA	Human	Primary HFF cells	RH	Microarray	No	Zeiner et al., 2010
Global miRNA expression profiling of domestic cat livers following acute *Toxoplasma gondii* infection	miRNA	Cat	Liver from infected cats	PRU	High-throughput sequencing	No	Cong et al., 2017
MicroRNA-132 dysregulation *in Toxoplasma gondii* infection has implications for dopamine signaling pathway	miRNA	Human	SK-N-MC cells^e^	RH, PRU, CTG	Microarray	No	Xiao et al., 2014
miR-146a and miR-155 delineate a MicroRNA fingerprint associated with *Toxoplasma* persistence in the host brain	miRNA	Human	Primary HFF	RH, ME49	Microarray	Yes	Cannella et al., 2014
*Toxoplasma* Modulates Signature Pathways of Human Epilepsy, Neurodegeneration and Cancer	miRNA	Human	S-NSC, S-NDC, MM6 cells^f^; Serum samples from ill children	GT1, PRU, ME49, VEG	High-throughput sequencing; qRT-PCR panel	No	Ngô et al., 2017
Comparison of splenocyte microRNA expression profiles of pigs during acute and chronic toxoplasmosis	miRNA	Pig	Spleen from infected pigs	YZ-1 (Chinese 1)	High-throughput sequencing	No	Hou et al., 2019
Expression profile of microRNAs in porcine alveolar macrophages after *Toxoplasma gondii* infection	miRNA	Pig	3D4-21 cell line^g^	RH, ME49	High-throughput sequencing	No	Li et al., 2019
Microarray analysis of long non-coding RNA expression profiles uncovers a *Toxoplasma*-induced negative regulation of host immune signaling	lncRNA	Human	HFF cell line	ME49	Microarray	Yes	Liu et al., 2018
*Toxoplasma gondii* manipulates expression of host long non-coding RNA during intracellular infection	lncRNA	Mouse	Bone marrow-derived macrophages	RH, PTG	Microarray	No	Menard et al., 2018

a *Tachyzoites used unless otherwise stated*.

b *Functional data*.

c *Human foreskin fibroblasts*.

d *Peripheral blood mononuclear cells*.

e *Human neuroepithelial cell line*.

f *S-NSC are adult human neural stem/progenitor cells; S-NDC are a differentiated form of S-NSC; MM6 are in vitro culture-adapted human monocytes*.

g *alveolar macrophages*.

### miR-17-92 Gene Cluster

The first study profiling microRNAs differentially expressed during *T. gondii* infection was published almost a decade ago (Zeiner et al., 2010). It reported that 14% of the miRNAs on the microarray were differentially regulated, including members of the miR-17-92 and miR-106b-25 family that are upregulated after infection with RH in primary human foreskin fibroblasts (HFFs). Other studies have confirmed this finding in other cell types. The miR-17-92 polycistronic gene cluster was also found to be upregulated in human macrophages derived from peripheral blood mononuclear cells after infection with TgCtwh3 strain parasites (Cai et al., 2013, 2014). He et al. found that mir-17-5p (a member of miR-17-92 gene cluster) is upregulated in the mouse spleen after infection with the RH strain (He et al., 2016). Nevertheless, in an initial analysis knockdown of miR-17-92 in HFF had no observable effect on infection, parasite replication or host cell lysis (Zeiner et al., 2010).

**Figure 2:**
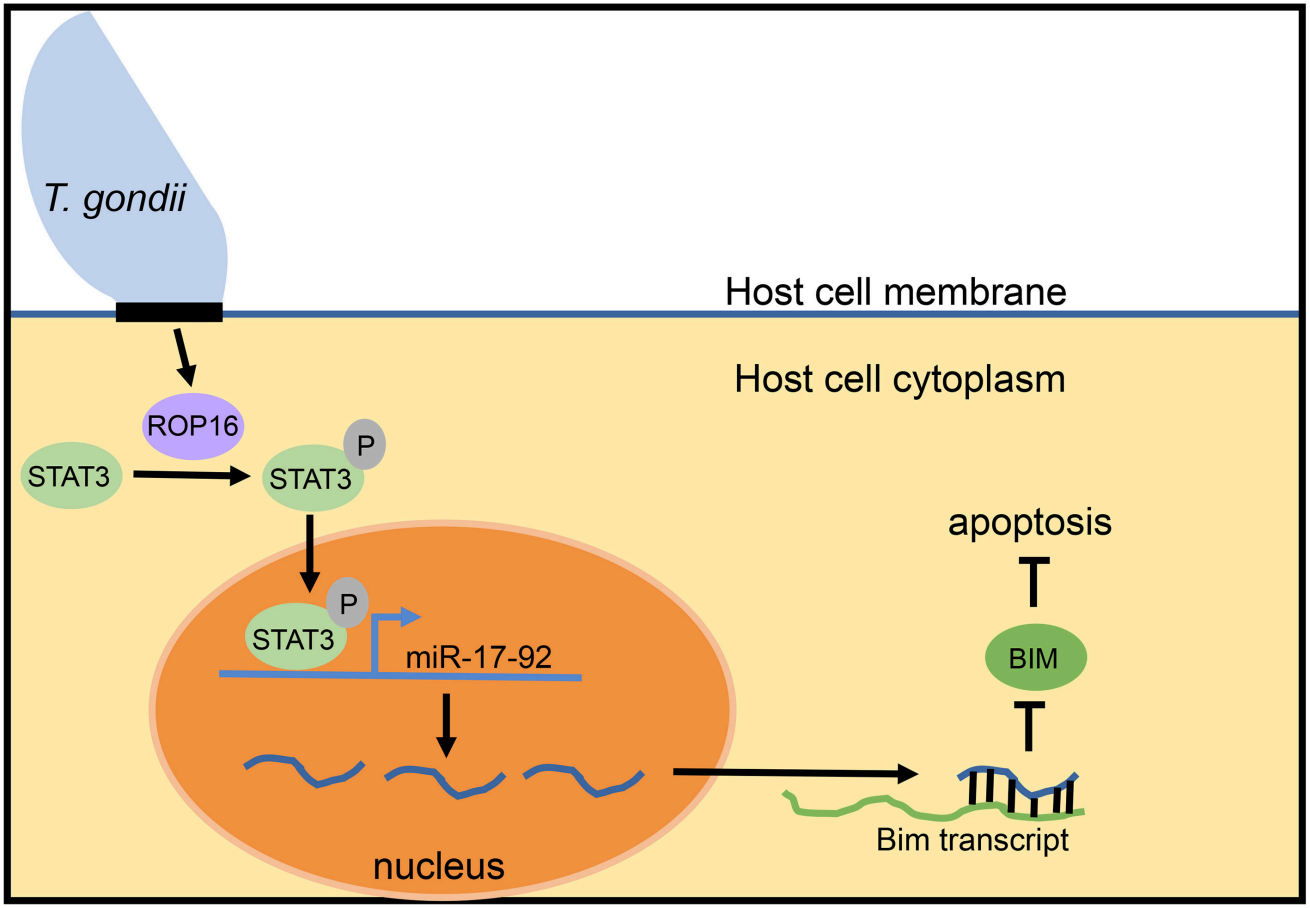
Model for mir-17-92 inhibition of apoptosis during *T. gondii* infection. During intracellular infection, *T. gondii* injects the ROP16 protein into the host cell cytoplasm. In the cytoplasm, ROP16 phosphorylates STAT3. Phosphorylated STAT3 enters the nucleus of the host cell, binds to STAT3 sites in the promoter of the miR-17-92 cluster gene, and upregulates miR-17-92 miRNAs. miR-17-92 cluster miRNAs then bind to the Bim transcript and reduce BIM levels, thereby inhibiting the process of apoptosis. This model is based upon data reported in Cai et al. (2013, 2014).

Despite lack of an overt effect of miR-17-92 on the intracellular growth cycle of *T. gondii*, more recent work suggests a connection of this miRNA cluster with inhibition of host cell apoptosis. Blocking apoptosis is a well-documented survival strategy of *Toxoplasma* in the host cell (Lüder and Gross, 2005; Carmen and Sinai, 2007). Knocking down expression of miR-20a (a member of the miR-17-92 cluster) in infected macrophages resulted in increased sensitivity to apoptosis (Cai et al., 2013; Rezaei et al., 2018). The model proposed for how *Toxoplasma* induces miR-17-92 to inhibit apoptosis revolves around the parasite secretory kinase ROP16 (Figure 2). This protein is injected into the host cell cytoplasm where it tyrosine phosphorylates STAT3 (Saeij et al., 2007; Yamamoto et al., 2009). The miR-17-92 cluster contains STAT3 binding sites in its promoter and is therefore upregulated by STAT3 phosphorylation. Evidence for this comes from the observation that knockdown of STAT3 using siRNA prevents miR-17-92 upregulation. Furthermore, luciferase constructs with the binding site for STAT3 in the promoter region of miR-17-92 led to increased luciferase expression (Cai et al., 2013). STAT3-induced miR-17-92 then binds to the 3′ UTR of the Bim transcript, reducing BIM levels (Cai et al., 2014). The BIM protein is pro-apoptotic and, therefore, deficiency leads to the inhibition of apoptosis observed in host cells following infection with *T. gondii*.

### miR-132

The miRNA miR-132 has both neural and immune functions and dysregulation has been associated with numerous neurological disorders (Soreq and Wolf, 2011; Miller et al., 2012; Wanet et al., 2012). Xiao et al. surveyed miRNA expression profiles in neural cells (SK-N-MC cells) acutely infected with Type I, Type II, and Type III *Toxoplasma* strains (Xiao et al., 2014). They found that miR-132 was the only miRNA upregulated by >2-fold by all three *T. gondii* strains, and this upregulation was confirmed in the peritoneal cavity and striatum of infected mice. Among the predicted targets of miR-132, the strongest pathway enriched was that for dopamine signaling. Three genes (Drd1, Drd5, and Maoa) in the dopamine signaling pathway displayed decreased transcription and protein expression in *T. gondii*-infected mice. HPLC data also demonstrated increased production of dopamine, serotonin, and 5-hydroxyindoleacetic acid in the striatum of *Toxoplasma*-infected mice. miR-132 is known to be upregulated by LPS and several viruses (Taganov et al., 2006; Lagos et al., 2010). These combined results suggest that miR-132 may be a common target of a broad range of pathogens and may represent a general response to infection. It is also worth noting that other researchers have reported dysregulation of dopamine pathways during *Toxoplasma* infection, suggesting possible links to nervous system abnormalities (Syn et al., 2018; Alsaady et al., 2019).

In a recent study, expression of miR-132 in the mouse brain during chronic (5 month) infection with PRU strain parasites was investigated (Li et al., 2015). Contrary to acute infection, chronic infection resulted in decreased expression of miR-132 relative to non-infected mice. Correlation between levels of miR-132 in different brain regions and the number of parasites was weak, suggesting that the effects of *Toxoplasma* on miRNA expression were indirect.

### miR-146a and miR155

Cannella et al. surveyed microRNAs differentially expressed between *T. gondii* high and low virulence strains to determine if differences in host pathogenesis correlated with specific microRNAs (Cannella et al., 2014). This resulted in identification of miR-146a and miR155. These investigators found that miR-146a was induced by Type II but not by Type I tachyzoites in several non-hematopoietic cell types. In addition, levels of miR-146a increased in the central nervous system of mice chronically infected with Type II *Toxoplasma* and were correlated with the presence of cysts. An independent study also found miR-146a was induced in the brain during chronic infection initiated with PRU oocysts (Hu et al., 2018).

In contrast to Type II *T. gondii*, Type I *Toxoplasma* strains were found to repress miR-146a levels. Furthermore, when ROP16 was deleted in Type I strains, expression of miR-146a was greatly enhanced. ROP16 deletion in Type II strains had no effect on miR-146a expression as predicted given the lack of kinase activity of this rhoptry protein. Interestingly, miR-146a^−/−^ mice displayed increased resistance to Type II *T. gondii* (Cannella et al., 2014). The lower levels of IFN-γ in miR-146a knockout mice suggests that increased resistance results from avoidance of cytokine-induced inflammation that can lead to pathology and death when not appropriately controlled (Gazzinelli et al., 1996; Gavrilescu and Denkers, 2001; Mordue et al., 2001).

The miRNA miR-155 is known to target SOCS1 mRNA to promote inflammation (Rao et al., 2014). Cannella et al. reported that miR-155 was induced after *T. gondii* infection (Cannella et al., 2014). Induction was strain-independent during *in vitro* infection of stromal and phagocytic cells. *In vivo*, miR-155 was induced in the central nervous system of mice infected with Type II *Toxoplasma* strains. Another study found that miR-155 was induced in mouse spleens after infection with RH tachyzoites (He et al., 2016). However, it is worth noting that in a study in pigs, miR-155 was downregulated during chronic infection (Hou et al., 2019). The reason for this conflicting result is not clear and could be due to host species or *T. gondii* strain differences. Nevertheless, taken together the data indicate that both miR-146a and miR-155 are triggered by *Toxoplasma*. At least for the case of miR-146a, this appears to impact the outcome of infection.

### MicroRNAs and Exosomes

Parasites exploit exosomes to influence host responses during infection (Gavinho et al., 2018; Wu et al., 2018; Atayde et al., 2019). It is, therefore, of interest that HFF infected with *T. gondii* release exosome-like vesicles containing several distinct miRNA species (Pope and Lässer, 2013). Thus, 10 miRNAs (miR-92a, miR-595, miR125b, miR-199a-3p, miR-125a-5p, miR-503, miR-320d, miR-1183, miR-99a-star, miR-23b) were identified within exosomes isolated from infected cells. The miRNA miR-23b is particularly of interest because it is a known negative regulator of IL-17 synthesis (Hu and O'Connell, 2012; Zhu et al., 2012). A more recent study identified as many as 64 distinct microRNAs that were present within exosomes derived from cells infected with RH vs. exosomes derived from uninfected cells (Kim et al., 2016). Additionally, the Cannella et al. (2014) study identified expression of a specific miRNA, miR-146a, in exosomes of Type II infected cells. Together, these data suggest that nearby uninfected cells (and not just infected cells) experience altered microRNA expression during infection. The influence of miRNA on exosome infection biology is likely to be a fruitful area of investigation in the near future.

### Host lncRNAs in the Response to *Toxoplasma*

To date, we know very little regarding expression of host long non-coding RNAs during *Toxoplasma* infection. Here, we highlight two studies that provide some interesting first glances at regulation of this class of RNA during *T. gondii* infection. While these studies are intriguing, direct comparison of the results is complicated by the small degree of sequence conservation between lncRNAs of different species (Spurlock et al., 2016).

### lncRNA Responses in Human Cells

One group of investigators examined lncRNAs differentially regulated in HFF cell lines during infection with Type II (ME49) *T. gondii* (Liu et al., 2018). They found that 1,206 lncRNAs were upregulated after infection, including 996 that were only induced by active infection relative to heat-inactivated parasites. Interestingly, inactivated parasites upregulated 392 lncRNAs when compared to uninfected cells, demonstrating that simply the presence of dead parasites can elicit effects on lncRNA expression. It was found that one particular lncRNA, NONHSAT022487, is highly upregulated after Type II infection. Knock down of NONHSAT022487 resulted in increased expression of cytokines IL-12, TNF-α, IL-1β, and IFN-γ in *T. gondii*-infected THP-1 monocytic cells. Overexpression of NONHSAT022487 resulted in decreased expression of these four cytokines. This lncRNA would therefore appear to be a prime candidate to further explore how it exerts its effects on proinflammatory cytokine gene expression.

### lncRNAs Regulated in Mouse Cells

We surveyed the expression of lncRNA in mouse bone marrow-derived macrophages during infection with both Type I (RH) and Type II (PTG) strains (Menard et al., 2018). We found that hundreds of putative lncRNAs were differentially regulated, and a substantially greater number were differentially regulated with RH rather than PTG infection (1,522 and 528, respectively). The total number of lncRNAs upregulated by infection was similar to the number down-regulated. Interestingly, we also found that *Toxoplasma* likely directly controls expression of host lncRNAs, as upregulation of two lncRNAs was ablated in parasite strains deleted for the STAT3/6-directed kinase ROP16. Functional studies are now required to identify biologically important lncRNAs in the response to infection.

## Conclusions

We have only a nascent understanding of the role of non-coding RNA in host defense against infection. However, we now know that there is a large number of miRNA that are up or downregulated during *T. gondii* infection, including the miR-17-92 gene cluster, miR-132, miR-146a, miR-155, and miR-23b. At least some of these miRNAs play important roles in the response to *T. gondii*. Clearly, there are many miRNA yet to be characterized, and it is likely that some will prove to significantly impact host defense largely in ways yet to be discovered. Our knowledge of lncRNA responses to *Toxoplasma* is even more rudimentary. However, the finding that thousands of lncRNAs that are up or downregulated during *T. gondii* infection indicates that host lncRNA responses are extensive, and most likely biologically important. Future work is required to determine those regulatory RNAs that are paramount in the response to infection with *Toxoplasma* and other microbial pathogens.

## Author Contributions

All authors listed have made a substantial, direct and intellectual contribution to the work, and approved it for publication.

### Conflict of Interest Statement

The authors declare that the research was conducted in the absence of any commercial or financial relationships that could be construed as a potential conflict of interest.
